# Retail Marketing of Menthol Cigarettes in Los Angeles, California: a Challenge to Health Equity

**DOI:** 10.5888/pcd18.200144

**Published:** 2021-02-11

**Authors:** Sabrina L. Smiley, Junhan Cho, Kacie C.A. Blackman, Tess Boley Cruz, Mary Ann Pentz, Jonathan M. Samet, Lourdes Baezconde-Garbanati

**Affiliations:** 1 Tobacco Center of Regulatory Science, Institute for Health Promotion and Disease Prevention Research, Department of Preventive Medicine, Keck School of Medicine, University of Southern California, Los Angeles, California; 2 Department of Health Sciences, Health Equity Research and Education Center, California State University, Northridge, California; 3 Norris Comprehensive Cancer Center, University of Southern California, Los Angeles, California; 4 Colorado School of Public Health, Aurora, Colorado

## Abstract

**Introduction:**

Sales of menthol cigarettes continue to increase, accounting for a third of the US cigarette market. Retail marketing of menthol cigarettes is a contributing factor to tobacco-related health disparities. To inform regulation to address associated disparities, we examined retail marketing strategies for menthol cigarettes and their features and characteristics in relation to neighborhood racial/ethnic composition.

**Methods:**

We used multilevel regression models to examine associations of neighborhood racial/ethnic composition and store type with menthol cigarette sales outcomes, including availability, exterior advertising, price promotions, and price in a sample of tobacco retailers (N = 673) in Los Angeles County neighborhoods with a median or below-median household income. We also recorded the prices of Newport cigarettes (the highest selling menthol cigarette brand in the United States) and blu disposable menthol e-cigarettes.

**Results:**

Overall, 94.5% of retailers sold menthol cigarettes, 31.2% displayed menthol cigarette price promotions, and 30.2% displayed at least one menthol cigarette advertisement on their exterior. Adjusting for racial/ethnic zip code cluster and store type, stores located in predominantly African American neighborhoods showed significantly higher odds in the availability of Newport cigarettes than stores in Hispanic neighborhoods (OR = 0.21; 95% CI, 0.09–0.53; *P* = .001) or non-Hispanic White (OR = 0.12; 95% CI, 0.05–0.31; *P* < .001) neighborhoods. Stores located in predominantly African American neighborhoods displayed significantly higher odds of having price promotions for menthol cigarettes and storefront advertisements than those in Hispanic neighborhoods (OR = 0.51; 95% CI, 0.30–0.88; *P* = .02 and OR = 0.25; 95% CI, 0.13–0.48; *P* < .001, respectively).

**Conclusion:**

In 2016 and 2017, menthol cigarettes were widely available in Los Angeles County across racial/ethnic neighborhoods. We found a disproportionate number of storefront advertisements and price promotions for menthol cigarettes in stores located in predominantly African American neighborhoods along with the lowest advertised pack price. This evidence supports tobacco control policies that restrict menthol cigarette sales in the retail environment.

**Summary**

**What is already known about this topic?**

Previous research shows that menthol cigarettes contribute to tobacco-related racial/ethnic health disparities.

**What is added by this report?**

To inform novel policy strategies restricting sales of menthol cigarettes and other flavored tobacco products in the retail environment, our study investigated whether retail marketing strategies for menthol cigarettes differed by neighborhood racial/ethnic composition (ie, African American, Korean American, Hispanic, non-Hispanic White) in Los Angeles County.

**What are the implications for public health practice?**

Retail marketing of menthol cigarettes was highest among stores in predominantly African American neighborhoods. Findings underscore the need to account for racial/ethnic neighborhood differences when developing, implementing, and evaluating novel policy strategies restricting menthol cigarette sales.

## Introduction

Menthol as a characterizing flavor in cigarettes is a continuing challenge to health equity. Menthol cigarette sales accounted for 36% of the US cigarette market in 2017, an upsurge from 26% in 2015 (1). As sales of nonmenthol cigarettes steadily decline (1), the increase in menthol cigarette sales is consistent with longitudinal data documenting the rise in past-30–day use of menthol cigarettes from 2002 through 2014, among non-Hispanic White, Asian, and Hispanic smokers (2). African Americans have the highest percentage of menthol cigarette use among all racial/ethnic groups, nearly 90% (3). Additionally, past-30–day use of menthol cigarettes is higher among adolescent smokers aged 12 to 17 years (56.7%) than among young adult smokers aged 18 to 25 (45%) or smokers 26 years or older (30.5%–34.7%) (3). Menthol cigarettes are considered more appealing than nonmenthol cigarettes, particularly by novice smokers, in part because of the anesthetic effects of the menthol flavor additive, which reduces harshness and improves the taste of cigarette smoke (4). For alternatives to cigarettes, smokers are also attracted to the appealing characteristics of e-cigarettes, including menthol-flavored types (5–7).

Evidence from the retail environment indicates that neighborhood demography influences retailer location and tobacco marketing (8,9). The 1998 Master Settlement Agreement and the 2009 Family Smoking Prevention and Tobacco Control Act (Tobacco Control Act) resulted in significant restrictions on tobacco industry marketing activities aimed at youth, such as prohibiting advertising of cigarettes and smokeless tobacco and the distribution of free samples of tobacco products (1,10,11). In response, retail settings like neighborhood convenience stores became major channels for the tobacco industry to market both menthol and nonmenthol cigarettes (1). For example, in 2017, the tobacco industry spent more than 90% of its $8.64 billion cigarette marketing budget on retail advertising and promotion, such as consumer coupons, price discounts to retailers, and shelving displays (1). Price discounts to retailers accounted for more than 70% ($6.18 billion) of the tobacco industry’s cigarette marketing budget in 2017 (1). Furthermore, spending on exterior advertising of cigarettes, including signage placed on storefronts, increased from $1.7 million in 2016 to $1.8 million in 2017 (1).

Exposure to retail tobacco marketing is a risk factor for smoking initiation among adolescents (12,13) and increased smoking among adults (9,14). The literature (15–17) on tobacco marketing disparities in the retail environment is growing, and findings indicate that low-income and majority non-White neighborhoods have high densities of tobacco retailers and are disproportionately exposed to marketing of cheap, harmful, combustible tobacco products, including menthol cigarettes. In a national sample of tobacco retail outlets, Mills et al (16) found reduced pricing of the Newport brand (manufactured by R.J. Reynolds) in neighborhoods with a high proportion of youths, African American residents, and low-income households. Newport is the highest selling menthol cigarette brand in the United States and the second-largest selling cigarette brand (18).

The 2009 Tobacco Control Act restricted all flavorings in cigarettes except menthol. The Act also granted the Food and Drug Administration (FDA) authority to regulate the manufacture, distribution, and marketing of tobacco products, including the authority to extend restrictions on flavored cigarettes to include menthol cigarettes (11). As a result of federal inaction, local jurisdictions (eg, Minneapolis, San Francisco) have themselves limited or restricted sales of menthol cigarettes and other flavored tobacco products (19). In June 2017, San Francisco became the first city to pass an ordinance that restricts the sale of any flavored tobacco product, including menthol, within the city limits (20). With growing local momentum coupled with increasing scientific evidence documenting disparities in menthol cigarette marketing and use (eg, the Tobacco Products Scientific Advisory Committee’s report on menthol ([21]), FDA in November 2018 announced its intent to remove menthol cigarettes from the market (22). However, the agency has yet to act.

In a unanimous vote on September 24, 2019, the Los Angeles County Board of Supervisors approved an ordinance establishing restrictions on retail sales of menthol cigarettes and other flavored tobacco products in unincorporated areas of the county (23). The ordinance became enforceable on May 1, 2020 (23). In August 2020, California’s Governor Gavin Newsom signed Senate Bill 793 to end the sale in the state of flavored tobacco products, including menthol cigarettes but excluding premium cigars, hookah, and some pipe tobacco (24). The law was set to go into effect on January 1, 2021 (25). However, state officials agreed to delay the effective date after opponents led by tobacco companies petitioned to bring the pending law to a statewide vote in 2022 (25).

Given the evolving landscape of menthol cigarette regulations, recent evidence on retail marketing of menthol cigarettes can help inform local ordinances in addition to the pending state laws to restrict the sale of menthol cigarettes. Therefore, we examined retail marketing strategies for menthol cigarettes and their association with neighborhood racial/ethnic composition in Los Angeles County, one of the largest and most diverse US counties. Our data were collected before the Los Angeles County ordinance was passed and before Governor Newsom signed Senate Bill 793.

## Methods

### Sample and procedures

We classified licensed tobacco retail stores in Los Angeles County into 6 categories: 1) small, independent convenience stores with or without a gas station; 2) beer, wine, and liquor stores; 3) small, independent grocery stores that primarily sold food; 4) tobacco-focused stores; 5) discount stores; and 6) other stores, such as donut shops and gas kiosks. We excluded pharmacies, big chain markets and supermarkets, and vape shops. Research has shown that independent and small licensed tobacco retailers use more tobacco advertising (26).

We selected stores in 2 steps. In step 1, zip codes with an annual median household income of $55,909 or below the median household income for Los Angeles County were ranked by percentage of races and ethnicities (27). The number of zip codes with residents predominantly non-Hispanic White, Hispanic, African American, or Korean American differed (non-Hispanic White, 32 zip codes; Hispanic, 14 zip codes; African American, 14 zip codes; Korean American, 7 zip codes). To be consistent across all racial/ethnic groups studied, we selected up to 15 zip codes available from each identified group. This criterion mostly affected the non-Hispanic White resident sample, which had 32 eligible zip codes available. All other racial/ethnic groups of focus had fewer than 15 zip codes that met the criterion. Therefore, we kept all zip codes in those groups. We collected data from the first 15 zip codes in the non-Hispanic White group and repeated that process until we reached our desired sample of 21 zip codes out of the possible 32 zip codes. From the 296 zip codes in Los Angeles County that had licensed tobacco retailers, we collected data for this study from 56 zip codes (19%).

In step 2, we randomly selected stores from ranked zip codes by using a comprehensive list of approximately 11,600 licensed tobacco retailers in Los Angeles County that is maintained by the California Department of Tax and Fee Administration (28). The number of stores selected was in proportion to the ranking by percentage of residents by race/ethnicity in each zip code. Store type was categorized by using standard definitions (29,30). Approximately 10,200 of the 11,600 licensed tobacco retailers were eligible under our store criteria, and 2,556 of the eligible stores were in the selected zip codes for our study (22% of licensed tobacco retail stores in Los Angeles County). We randomly selected a total of 1,480 licensed tobacco retailers; 310 were deemed ineligible on the basis of the above inclusion criteria. We visited 1,170 eligible stores with the goal of conducting 700 in-person store observations. Of the 1,170 stores visited, 831 (71%) agreed to participate. We selected 700 of the 831 for our sample. Of the 700 selected, 679 were licensed tobacco retailers who allowed an observation; however, because of missing data, only 673 of these were included in our study. We estimated that our sample represented 21% of the licensed tobacco retailers that sold tobacco for all communities in the zip codes selected and 6% of all licensed tobacco retailers in Los Angeles County (28). Our sampling design process is described in detail elsewhere (27). The Institutional Review Board of the University of Southern California approved the study (HS#13–00647).

### Data collection

We collected data from participating stores from January 2016 through April 2017. We used a store audit checklist adapted from the Standardized Tobacco Assessment for Retail Settings observation tool (31). Nineteen community health workers, including *promotores de salud*, participated in training to conduct the store observations and take digital photographs of each store’s exterior and interior. This in-person training consisted of a detailed protocol for recording exterior and interior store observations of tobacco products and marketing materials and supervised practice field work. Store observations were completed by community health workers in zip codes with a high percentage of residents of the following races/ethnicities: African American (194 stores), non-Hispanic White (193 stores), Hispanic (187 stores), and Korean American (99 stores). Respondents representing their retail shop consented to permit the store observation, and those who agreed received a $50 gift card and a printed information packet (available in English, Spanish, and Korean) containing fact sheets about the FDA’s tobacco regulatory authority.

### Measures

Community health workers coded the marketing and advertising of menthol cigarettes in 4 domains: 1) availability, 2) exterior advertisements, 3) price promotions, and 4) price. Availability was assessed by a yes or no answer to the following questions: Any cigarettes sold here? Are menthol cigarettes sold here? Are Newport cigarettes sold here? Availability of blu menthol disposable e-cigarettes was also assessed by a yes or no answer to the following question: Are blu menthol disposable e-cigarettes sold here? Storefront exterior advertising was assessed with yes or no to the following inquiries: Are menthol cigarettes advertised outside the store? Are nonmenthol cigarettes advertised outside the store? Price promotions were coded by location (interior/exterior), defined to include any multipack special (eg, buy one get one free) or special price (eg, $1.00 off) and assessed by the presence or absence (yes or no) of any cigarette price promotions or any menthol cigarette price promotions. To assess price, community health workers recorded the lowest advertised single-pack price for cigarettes, for Newport menthol cigarettes, and for blu menthol disposable e-cigarettes. Interrater reliability was assessed at 210 stores. Cohen κ statistics for binary measures ranged from 0.59 for menthol cigarette price promotions to 0.94 for availability of cigarettes. Good reliability was obtained for cigarette prices (minimum intraclass correlation coefficient [ICC] = 0.71 for a pack of Newport menthol cigarettes, maximum ICC = 0.90 for the cheapest cigarette pack, and ICC = 0.67 for blu menthol disposable e-cigarettes).

### Data analysis

We used frequency distributions and cross tabulations for descriptive statistics of store type and product availability, exterior advertisements, price promotions, and price by racial/ethnic zip code cluster. Descriptive statistics were also computed to characterize product availability, exterior advertisements, price promotions, and price by store type. We then examined associations of racial/ethnic zip code cluster and store type with outcomes of marketing menthol cigarettes and related tobacco products. To identify independent and relative effects of neighborhood-level and store-level factors, we conducted regression tests in both unadjusted and adjusted models: univariable models included each individual regressor and multivariable models included both neighborhood and store regressors. Hypotheses were tested by using multilevel regression modeling implemented in Mplus version 7 (Mplus). Because the stores were nested in zip codes, 2-level models were used to adjust parameter standard errors for interdependence in the data. Level 1 was defined as the store-level factor of store type, and level 2 was defined as the neighborhood-level factor of racial/ethnic zip code cluster. Multilevel regression modeling of binary outcomes yielded odds ratios (ORs) and 95% CIs with significance set at *P* < .05 (2-tailed) for binary logistic regression coefficients. Multilevel regression modeling for continuous outcomes (ie, price) were unstandardized regression coefficients. Missing data were managed with maximum likelihood estimation.

## Results

Convenience stores with or without gasoline sales (36% of our sample) were the most common store type, followed by small, independent grocery stores (28.2%), liquor stores (15.9%), tobacco-focused stores (9.5%), discount stores (6.5%), and other store types (4.2%). Nearly 95% of these stores sold menthol cigarettes, 87.7% offered Newport packs, and 20.8% offered blu menthol disposable e-cigarettes. Of the 673 stores, 35.2% had exterior advertisements for nonmenthol cigarettes, and 30.2% had exterior advertisements for menthol cigarettes. Approximately 30% of stores offered price promotions on packs of menthol cigarettes. The average price for the cheapest pack of menthol cigarettes was $5.00 (standard deviation [SD], 1.14). The average pack price for Newport cigarettes was $6.45 (SD = 0.78, n = 590 stores), and the average pack price for blu menthol disposable e-cigarettes was $10.10 (SD = 1.73, n = 139 stores).

The availability of Newport cigarettes was significantly (*P* < .001) more common in African American (95.9%) and Korean American (92.9%) neighborhoods (Table 1). Blu menthol disposable e-cigarettes were significantly (*P* < .001) more common in non-Hispanic White (32.6%) neighborhoods than in African American (19.1%), Korean American (18.2%), or Hispanic (11.8%) neighborhoods. Newport cigarettes cost significantly (*P* < .001) less per pack in African American neighborhoods ($6.19) than in non-Hispanic White ($6.53), Hispanic ($6.55), and Korean American ($6.66) neighborhoods.

**Table 1:** Product Availability, Exterior Advertisement, Price Promotions, and Price, by Race/Ethnicity Zip Code Cluster (N = 673), Los Angeles, California, 2016–2017

Variables, Menthol Cigarette Retail Marketing	Race/Ethnicity Zip Code Cluster				*P* Value, Group Differences
	African American (n = 194)	Non-Hispanic White (n = 193)	Hispanic/Latino (n = 187)	Korean American (n = 99)	
**Availability, n (%)**					
Cigarette, single pack	188 (96.9)	188 (97.4)	187 (100.0)	97 (98.0)	.14^a^
Menthol cigarette, single pack	184 (94.8)	183 (94.8)	175 (93.6)	94 (94.9)	.94^a^
Newport menthol cigarette, single pack	186 (95.9)	157 (81.3)	155 (82.9)	92 (92.9)	<.001^a^
Blu menthol disposable e-cigarette, single pack	37 (19.1)	63 (32.6)	22 (11.8)	18 (18.2)	<.001^a^
**Advertisement, n (%)**					
Exterior advertisement (nonmenthol cigarette)	79 (40.7)	85 (44.0)	40 (21.4)	33 (33.3)	<.001^a^
Exterior advertisement (menthol cigarette)	83 (42.8)	64 (33.2)	29 (15.5)	27 (27.3)	<.001^a^
**Price promotion, n (%)**					
Cigarette price promotion	76 (39.2)	92 (47.7)	43 (23.0)	24 (24.2)	<.001^a^
Menthol cigarette price promotion	67 (34.5)	84 (43.5)	38 (20.3)	21 (21.2)	<.001^a^
**Price, $, mean (standard deviation)**					
Cheapest cigarette, single pack	5.43 (0.73)	5.82 (0.93)	5.81 (0.91)	5.77 (0.77)	<.001^b^
Cheapest menthol cigarette, single pack	4.68 (0.99)	5.21 (1.16)	5.16 (1.24)	4.93 (1.03)	<.001^b^
Newport menthol cigarette, single pack	6.19 (0.77)	6.53 (0.85)	6.55 (0.72)	6.66 (0.64)	<.001^b^
Blu menthol disposable e-cigarette, single pack	10.07 (2.01)	10.47 (1.76)	9.89 (0.84)	9.09 (1.50)	.02^b^

a Calculated by using χ^2^ test.

b Calculated by using 1-way analysis of variance (ANOVA).

We assessed the results for 8 outcomes: 1) any cigarettes sold, 2) any menthol cigarettes sold, 3) any Newport cigarettes sold, 4) any blu menthol disposable e-cigarettes sold (Table 2), 5) any cigarette price promotions, 6) any menthol cigarette price promotions, 7) any nonmenthol cigarette exterior advertisements, and 8) any menthol cigarette exterior advertisements (Table 3). Stores located in neighborhood clusters with predominantly African American residents had significantly higher odds of selling Newport cigarettes than stores located in neighborhood clusters with predominantly Hispanic (OR = 0.21; 95% CI, 0.09–0.47; *P* < .001) or non-Hispanic White (OR = 0.19; 95% CI, 0.09–0.42; *P* < .001) residents (Table 2). After adjusting for racial/ethnic zip code cluster and store type simultaneously, the association persisted (non-Hispanic White, OR = 0.12; 95% CI, 0.05–0.31; *P* = .01; Hispanic, OR = 0.21, 95% CI, 0.09–0.53; *P* < .001). Stores located in neighborhood clusters with predominantly African American residents had significantly lower odds of selling blu menthol disposable e-cigarettes than stores located in neighborhood clusters with predominantly non-Hispanic White (OR = 2.06; 95% CI, 1.29–3.28; *P* = .003) or Hispanic residents (OR = 0.57; 95% CI, 0.32–1.01; *P* = .05). The association was nonsignificant after adjusting for racial/ethnic zip code cluster and store type (OR = 1.62; 95% CI, 0.96–2.72; *P* = .07 and OR = 0.67, 95% CI, 0.37–1.22; *P* = .19, respectively).

**Table 2:** Associations Between Race/Ethnicity Zip Code Cluster (N = 673) and Store Type and Product Availability, Los Angeles County, California, 2016–2017

Regressors	Menthol Cigarette Retail Marketing Outcomes, OR (95% CI) [*P* Value]^a^			
	Any Cigarettes	Any Menthol Cigarettes	Newport Cigarettes	Blu Menthol Disposable E-Cigarettes
**Univariable model^b^**				
**Race/ethnicity zip code cluster**				
Black/African American	Reference			
Korean American	1.55 (0.31–7.81) [.60]	1.02 (0.34–3.08) [.97]	0.57 (0.20–1.61) [.29]	0.94 (0.51–1.76) [.85]
Hispanic	—	0.79 (0.33–1.88) [.60]	0.21 (0.09–0.47) [<.001]	0.57 (0.32–1.01) [.05]
Non-Hispanic White	1.20 (0.36–4.00) [.77]	0.99 (0.40–2.45) [.99]	0.19 (0.09–0.42) [<.001]	2.06 (1.29–3.28) [.003]
**Store type**				
Gasoline/convenience store	Reference			
Liquor store	1.82 (0.22–15.45) [.58]	2.28 (0.54–9.71) [.26]	0.53 (0.17–1.62) [.26]	0.40 (0.22–0.75) [.004]
Grocery store	1.60 (0.37–6.82) [.53]	0.59 (0.27–1.28) [.18]	0.15 (0.05–0.45) [.001]	0.09 (0.04–0.21) [<.001]
Discount store	0.70 (0.10–4.95) [.72]	0.44 (0.13–1.43) [.17]	0.20 (0.07–0.58) [.003]	0.27 (0.09–0.77) [.01]
Tobacco-focused store	0.20 (0.06–0.66) [.01]	0.42 (0.16–1.12) [.08]	0.21 (0.08–0.56) [.002]	1.41 (0.79–2.54) [.25]
Other^c^	—	0.67 (0.12–2.57) [.46]	0.11 (0.04–0.33) [<.001]	0.24 (0.07–0.87) [.03]
**Multivariable model^d^**				
**Race/ethnicity zip code cluster**				
Black/African American	Reference			
Korean American	1.45 (0.20–10.76) [.71]	1.01 (0.32–3.16) [.98]	0.61 (0.23–1.65) [.33]	1.10 (0.47–2.61) [.82]
Hispanic	—	0.78 (0.34–1.79) [.59]	0.21 (0.09–0.53) [.001]	0.67 (0.37–1.22) [.19]
Non-Hispanic White	1.41 (0.52–3.86) [.50]	0.93 (0.44–1.97) [.89]	0.12 (0.05–0.31) [<.001]	1.62 (0.96–2.72) [.07]
**Store type**				
Gasoline/convenience store	Reference			
Liquor store	1.84 (0.22–15.25) [.57]	2.26 (0.53–9.64) [.27]	0.57 (0.20–1.64) [.30]	0.40 (0.22–0.73) [.003]
Grocery store	1.34 (0.28–6.40) [.72]	0.61 (0.27–1.35) [.22]	0.13 (0.03–0.38) [<.001]	0.10 (0.04–0.24) [<.001]
Discount store	0.63 (0.08–5.07) [.66]	0.44 (0.13–0.51) [.19]	0.15 (0.05–0.43) [<.001]	0.32 (0.11–0.91) [.03]
Tobacco-focused store	0.24 (0.08–0.72) [.01]	0.41 (0.16–1.04) [.06]	0.25 (0.10–0.60) [.002]	1.25 (0.70–2.25) [.45]
Other^d^	—	0.56 (0.12–2.54) [.45]	0.13 (0.05–0.35) [<.001]	0.22 (0.06–0.82) [.02]

Abbreviations: OR = odds ratio; —, not applicable.

a Multilevel binary logistic regression models for each binary outcome. Values refer to single packs.

b Univariable models including individual race/ethnicity zip code cluster and store type regressor, separately. Unadjusted associations between each regressor and outcomes of retail marketing of menthol cigarettes are shown.

c Includes donut shop and gas kiosk.

d Multivariable model including race/ethnicity zip code cluster and store type regressors simultaneously. Adjusted associations between each regressor and outcomes of menthol cigarette retail marketing are shown.

**Table 3:** Associations Between Racial/Ethnic Zip Code Cluster and Store Type With Price Promotion and Exterior Advertisement, Los Angeles, California, 2016–2017

Regressors	Menthol Cigarette Retail Marketing Outcomes			
	Price Promotion, Any Cigarettes^a^	Price Promotion, Menthol Cigarettes^a^	Exterior Advertisement, Nonmenthol Cigarettes^a^	Exterior Advertisement, Menthol Cigarettes^a^
**Univariable model^b^**				
**Race/ethnicity zip code cluster**				
Black/African American	Reference			
Korean American	0.50 (0.29–0.86) [.01]	0.51 (0.29–0.90) [.02]	0.73 (0.44–1.21) [.22]	0.50 (0.30–0.85) [.01]
Hispanic	0.46 (0.30–0.72) [.001]	0.48 (0.30–0.77) [.002]	0.40 (0.25–0.62) [<.001]	0.25 (0.15–0.40) [<.001]
Non-Hispanic White	1.41 (0.95–2.12) [.09]	1.46 (0.97–2.20) [.07]	1.15 (0.77–1.72) [.51]	0.66 (0.44–1.01) [.05]
**Store type**				
Gasoline/convenience store	Reference			
Liquor store	0.35 (0.21–0.58) [<.001]	0.36 (0.20–0.63) [<.001]	0.24 (0.12–0.47) [<.001]	0.19 (0.10–0.37) [<.001]
Grocery store	0.13 (0.07–0.23) [<.001]	0.11 (0.05–0.22) [<.001]	0.15 (0.10–0.25) [<.001]	0.13 (0.07–0.24) [<.001]
Discount store	0.27 (0.14–0.53) [<.001]	0.17 (0.07–0.44) [<.001]	0.36 (0.18–0.72) [.004]	0.43 (0.20–0.93) [.03]
Tobacco-focused store	0.43 (0.24–0.80) [.007]	0.44 (0.26–0.77) [.004]	1.53 (0.84–2.79) [.17]	0.99 (0.51–1.92) [.97]
Other^c^	0.15 (0.06–0.43) [<.001]	0.14 (0.04–0.45) [.001]	0.64 (0.26–1.58) [.34]	0.18 (0.05–0.63) [.007]
**Multivariable model^d^**				
**Race/ethnicity zip code cluster**				
Black/African American	Reference			
Korean American	0.54 (0.21–1.36) [.19]	0.56 (0.25–1.25) [.16]	0.83 (0.38–1.83) [.64]	0.58 (0.28–1.20) [.14]
Hispanic	0.49 (0.28–0.86) [.01]	0.51 (0.30–0.88) [.02]	0.43 (0.21–0.88) [.02]	0.25 (0.13–0.48) [<.001]
Non-Hispanic White	1.33 (0.78–2.28) [.30]	1.28 (0.77–2.12) [.35]	0.90 (0.48–1.68) [.73]	0.54 (0.31–0.92) [.03]
**Store type**				
Gasoline/convenience store	Reference			
Liquor store	0.35 (0.21–0.58) [<.001]	0.36 (0.20–0.63) [<.001]	0.24 (0.12–0.46) [<.001]	0.19 (0.10–0.38) [<.001]
Grocery store	0.14 (0.08–0.25) [<.001]	0.12 (0.06–0.24) [<.001]	0.16 (0.10–0.26) [<.001]	0.14 (0.08–0.24) [<.001]
Discount store	0.31 (0.16–0.59) [<.001]	0.20 (0.08–0.50) [.001]	0.38 (0.19–0.74) [.004]	0.43 (0.20–0.91) [.03]
Tobacco-focused store	0.38 (0.20–0.71) [.002]	0.39 (0.23–0.68) [.001]	1.39 (0.76–2.54) [.29]	0.87 (0.46–1.67) [.68]
Other^c^	0.15 (0.05–0.43) [<.001]	0.14 (0.04–0.46) [.001]	0.62 (0.25–1.53) [.30]	0.17 (0.05–0.60) [.006]

a Multilevel binary logistic regression models for each binary outcome. Values are odds ratio (95% CI) [*P* value].

b Univariable models including individual racial/ethnic zip code cluster and store type regressor, separately. Unadjusted associations between each regressor and menthol cigarette retail marketing outcomes are shown.

c Includes donut shops and gas kiosks.

d Multivariable model including racial/ethnic zip code cluster and store type regressors simultaneously. Adjusted associations between each regressor and outcomes of menthol cigarette retail marketing are shown.

The odds of displaying a price promotion for menthol cigarettes were significantly higher in stores located in neighborhood clusters with predominantly African American residents than in stores located in neighborhood clusters with predominantly Hispanic (OR = 0.48; 95% CI, 0.30–0.77; *P* = .002) or Korean American residents (OR = 0.51; 95% CI, 0.29–0.90; *P* = .02) (Table 3). These associations were nonsignificant after adjusting for racial/ethnic zip code cluster and store type, except for stores located in neighborhood clusters with predominantly Hispanic residents (OR = 0.51; 95% CI, 0.30–0.88; *P* = .02). All stores had significantly lower odds of displaying at least 1 price promotion for menthol cigarettes compared with gasoline/convenience stores. These associations persisted after adjusting for racial/ethnic zip code cluster and store type simultaneously. The odds of a store displaying at least 1 exterior advertisement for menthol cigarettes were significantly higher in stores located in neighborhood clusters with predominantly African American residents than in stores located in predominantly Hispanic (OR = 0.25, 95% CI, 0.15–0.40; *P* < .001), Korean American (OR = 0.50; 95% CI, 0.30–0.85; *P* = .01), or non-Hispanic White (OR = 0.66; 95% CI, 0.44–1.01; *P* = .05) neighborhood clusters. These associations persisted after adjusting for racial/ethnic zip code cluster and store type, except for stores located in neighborhood clusters with a higher proportion of Korean American residents (OR = 0.58; 95% CI, 0.28–1.20; *P* = .14). After adjusting for racial/ethnic zip code cluster and store type, gasoline/convenience stores had significantly higher odds of displaying exterior advertisements for menthol cigarettes compared with liquor stores (OR = 0.19; 95% CI, 0.10–0.38; *P* < .001), grocery stores (OR = 0.14; 95% CI, 0.08–0.24; *P* < .001), and discount stores (OR = 0.43; 95% CI, 0.20–0.91; *P* = .03), but not tobacco-focused stores (OR = 0.87; 95% CI, 0.46–1.67; *P* = .68).

We assessed the cheapest single-pack price for the following products: any cigarettes, menthol cigarettes, Newport menthol cigarettes, and blu menthol disposable e-cigarettes (Table 4). Adjusting for racial/ethnic zip code cluster and store type, the price of the cheapest menthol cigarette single pack was significantly lower in stores located in African American neighborhoods compared with Hispanic (b = 0.39: 95% CI, 0.18–0.60; *P* < .001) and non-Hispanic White (b = 0.64; 95% CI, 0.33–0.96; *P* < .001) neighborhoods. The prices of both Newport menthol cigarette single-pack and cheapest cigarette single-pack were significantly lower in African American neighborhoods than in Korean American, Hispanic, and non-Hispanic White neighborhoods (*P* values, ≤ .008). For example, after adjusting for store type, the estimated price of a Newport single pack was $0.38 higher in non-Hispanic White neighborhoods (b = 0.38; 95% CI, 0.16–0.60; *P* = .001) than in African American neighborhoods.

**Table 4:** Associations Between Racial/Ethnic Zip Code Cluster (N = 673) and Store Type and Product Price, Los Angeles, California, 2016–2017

Regressors	Menthol Cigarette Retail Marketing Outcomes^a^			
	Blu Menthol Disposable E-Cigarettes	Newport Menthol Cigarettes	Cheapest Menthol Cigarettes	Cheapest Any Cigarettes
**Univariable model^b^**				
**Race/ethnicity zip code cluster**				
Black/African American	Reference			
Korean American	−0.98 (−1.92 to 0.04) [.04]	0.47 (0.28 to 0.66) [<.001]	0.25 (−0.03 to 0.52) [.08]	0.34 (0.13 to 0.54) [.002]
Hispanic	−0.18 (−1.07 to 0.70) [.68]	0.36 (0.19 to 0.52) [<.001]	0.48 (0.26 to 0.71) [<.001]	0.38 (0.21 to 0.55) [<.001]
Non-Hispanic White	0.40 (−0.28 to 1.09) [.25]	0.33 (0.17 to 0.50) [<.001]	0.53 (0.31 to 0.76) [<.001]	0.39 (0.22 to 0.56) [<.001]
**Store type**				
Gasoline/convenience store	Reference			
Liquor store	0.39 (−0.36 to 1.14) [.31]	0.11 (−0.08 to 0.30) [.26]	−0.08 (−0.36 to 0.19) [.55]	−0.03 (−0.24 to 0.19) [.81]
Grocery store	−1.07 (−2.25 to 0.12) [.08]	0.31 (0.15 to 0.47) [<.001]	0.50 (0.27 to 0.73) [<.001]	0.35 (0.18 to 0.53) [<.001]
Discount store	−0.25 (−0.91 to 0.42) [.47]	−0.09 (−0.28 to 0.10) [.35]	0.19 (−0.10 to 0.48) [.21]	0.01 (−0.20 to 0.20) [.98]
Tobacco-focused store	0.43 (−0.55 to 1.42) [.39]	−0.43 (−0.58 to 0.27) [<.001]	−0.49 (−0.78 to 0.20) [.001]	−0.50 (−0.71 to 0.29) [<.001]
Other^c^	0.62 (0.01 to 1.24) [.04]	0.42 (0.13 to 0.70) [.005]	1.21 (0.81 to 1.61) [<.001]	0.73 (0.39 to 1.07) [<.001]
**Multivariable model^d^**				
**Race/ethnicity zip code cluster**				
Black/African American	Reference			
Korean American	−1.14 (−2.02 to 0.26) [.01]	0.42 (0.24 to 0.60) [<.001]	0.20 (−0.03 to 0.42) [.08]	0.29 (0.12 to 0.46) [.001]
Hispanic	−0.17 (−1.01 to 0.66) [.69]	0.27 (0.07 to 0.47) [.008]	0.39 (0.18 to 0.60) [<.001]	0.28 (0.12 to 0.45) [.001]
Non-Hispanic White	0.22 (−0.72 to 1.15) [.65]	0.38 (0.16 to0.60) [.001]	0.64 (0.33 to 0.96) [<.001]	0.46 (0.21 to 0.71) [<.001]
**Store type**				
Gasoline/convenience store	Reference			
Liquor store	0.33 (−0.35 to 1.00) [.34]	0.11 (−0.08 to 0.29) [.27]	−0.07 (−0.35 to 0.20) [.60]	−0.03 (−0.24 to 0.19) [.82]
Grocery store	−0.76 (−2.23 to 0.71) [.31]	0.33 (0.17 to 0.49) [<.001]	0.54 (0.31 to 0.77) [<.001]	0.38 (0.20 to 0.56) [<.001]
Discount store	−0.26 (−2.21 to 1.68) [.79]	−0.06 (−0.24 to 0.13) [.54]	0.27 (−0.04 to 0.57) [.09]	0.05 (−0.17 to 0.26) [.66]
Tobacco-focused store	0.36 (−0.61 to 1.33) [.47]	−0.42 (−0.57 to 0.27) [<.001]	−0.50 (−0.78 to 0.21) [.001]	−0.50 (−0.71 to 0.29) [<.001]
Other^c^	1.25 (−0.23 to 2.73) [.10]	0.42 (0.12 to 0.71) [.006]	1.19 (0.80 to 1.60) [<.001]	0.72 (0.37 to 1.06) [<.001]

a Multilevel regression models for each continuous outcome. Values are b (95% CI) [*P* value] and refer to single packs.

b Univariable models including individual racial/ethnic zip code cluster and store type regressor, separately. Unadjusted associations between each regressor and menthol cigarette retail marketing outcomes are shown.

c Includes donut shops and gas kiosks.

d Multivariable model including racial/ethnic zip code cluster and store type regressors simultaneously. Adjusted associations between each regressor and outcomes of menthol cigarette retail marketing are shown.

## Discussion

Menthol cigarettes were widely available in Los Angeles County during our study period across racial/ethnic neighborhoods. Nearly all stores in our sample sold menthol cigarettes, and 87.7% sold Newport. This evidence supports tobacco control policies that restrict menthol cigarette sales in the retail environment. Notably, a disproportionate quantity of storefront advertisements, price promotions, and lowest advertised pack price for menthol cigarettes, including Newport, was found in stores located in predominantly African American neighborhoods. These findings align with previous research (16) that found more price promotions for Newport near areas with predominantly African American residents.

Nearly all stores in Korean American and Hispanic neighborhoods sold menthol cigarettes, including Newport, and at least 20% displayed a price promotion for menthol cigarettes. In recent years, population-based survey research (2) found an increase in current (past 30-day) menthol cigarette use from 2012–2014 among Hispanic (47%), Asian (38%), and non-Hispanic White (29%) smokers (aged ≥12), compared with 2008–2010 (37.1%, 30.3%, 26%, respectively). Study findings suggest that restrictions on menthol cigarettes and price promotions can lead to reductions in the prevalence of menthol cigarette use across subpopulations.

In contrast to Newport, blu menthol disposable e-cigarettes were more likely to be sold in tobacco-focused stores and gasoline/convenience stores located in neighborhoods with predominantly non-Hispanic White residents. Our study findings support recent evidence (32) on e-cigarette availability and advertising and variations by racial/ethnic neighborhood. For example, in New York City, Giovenco et al (32) found that e-cigarettes and smokeless tobacco were more accessible in predominantly non-Hispanic White and higher-income neighborhoods than in predominantly Black, Hispanic, and low-income neighborhoods. This combination of findings suggests a consistent retail marketing strategy for e-cigarettes in the United States. Additionally, this combination of findings could mean widening tobacco-related health disparities if combustible cigarette use persists in racial/ethnic minority neighborhoods while majority non-Hispanic White neighborhoods have increased access to e-cigarettes.

Our study has limitations. First, zip codes represent reasonably accurate racial/ethnic boundaries because of the relatively high level of residential segregation in Los Angeles; however, they do not always represent exact neighborhood boundaries and provide less granularity than census tracts. Second, findings are limited to low-income zip codes in Los Angeles County and may not be generalizable to the county as a whole, to other large urban areas in the United States, or to areas with less racial/ethnic diversity. Third, a limitation of studying prices for the leading brands of menthol cigarettes and e-cigarettes is that these prices reflect promotional strategies that are determined by different manufacturers (33). Nevertheless, study findings are consistent with national, state, and regional findings from retail surveys that showed that menthol cigarettes are more prevalent in areas with a high proportion of African American residents. Also, few studies have specifically examined retail marketing of menthol cigarettes in Korean American and Hispanic neighborhoods. Study findings add to a growing body of evidence that e-cigarettes are more accessible in predominantly non-Hispanic White neighborhoods. Other strengths of this study are a large representative sample of licensed tobacco retailers and a standardized data collection protocol (31).

As the sale of menthol cigarettes continues to increase each year (1), it is vital for governments —local, state, and federal — to pursue policies that eliminate menthol cigarette sales and regulate the retail environment. Our study provides new information regarding racial/ethnic neighborhood disparities in retail marketing of menthol cigarettes, which can inform the pending law (SB793) in California (24,25) and provides an argument for the enforcement of existing regulations in the unincorporated areas of Los Angeles County (23). Future research is needed to include resident and retailer perceptions of retail marketing of menthol cigarettes and policies to restrict menthol cigarette sales. Our data also add novel information regarding marketing of menthol cigarettes and e-cigarettes to Korean American and Hispanic communities and contribute to existing evidence (9,12–14) that retail marketing of menthol cigarettes is a contributing factor to disproportionate use among African American smokers. The retail environment is the dominant channel for marketing tobacco products (1), and documenting marketing strategies for menthol cigarettes can inform regulation that reduces racial/ethnic disparities in access to menthol cigarettes and resultant tobacco-related morbidity and mortality.
